# High retention in an opioid agonist therapy project in Durban, South Africa: the role of best practice and social cohesion

**DOI:** 10.1186/s12954-020-00368-1

**Published:** 2020-04-15

**Authors:** Monique Marks, Andrew Scheibe, Shaun Shelly

**Affiliations:** 1 Urban Futures Centre, Steve Biko Campus, Durban University of Technology, Durban, South Africa; 2 TB HIV Care, 7th Floor, 11 Adderley Street, Cape Town, South Africa; 3 Department of Family Medicine, University of Pretoria, Pretoria, South Africa

**Keywords:** Low threshold, Heroin, Psychosocial integration, Group intervention, Peer support, Social cohesion

## Abstract

**Background:**

Moral conservatism within government and communities has resulted in a reluctance to support the provision of opioid agonist therapy for people with opioid use disorders in South Africa. In April 2017, South Africa’s first low-threshold opioid agonist therapy demonstration project was launched in Durban. The project provided 54 low-income people with heroin use disorders methadone and voluntary access to psychosocial services for 18 months. At 12 months, retention was 74%, notably higher than the global average. In this paper, we aim to make sense of this outcome.

**Methods:**

Thirty semi-structured interviews, two focus groups, ten oral histories and ethnographic observations were done at various project time points. These activities explored participants’ pathways into drug use and the project, their meaning attributed to methadone, the factors contributing to project success and changes they experienced. Recordings, transcripts, notes and feedback were reviewed and triangulated. Key factors contributing to retention were identified and analysed in light of the existing literature.

**Results:**

The philosophy and architecture of the project, and social cohesion were identified as the main factors contributing to retention. The use of a harm reduction approach enabled participants to set and be supported to achieve their treatment goals, and was shown to be important for the development of trusting therapeutic relationships. The employment of a restorative justice paradigm provided a sense of acceptance of humanity and flaws as well as an imperative to act responsibly towards others, fostering a culture of respect. Social cohesion was fostered through the facilitation of group sessions, a peace committee and group sport (soccer). In concert, these activities provided opportunities for participants to demonstrate care and interest in one another’s life, leading to interdependence and care, contributing to them remaining in the project.

**Conclusions:**

We believe that the high retention was achieved through attraction. We argue that opioid agonist therapy programmes should take the principles of harm reduction and restorative justice into consideration when designing low-threshold opioid agonist therapy services. Additionally, ways to support cohesion amongst people receiving agonist therapy should be explored to support their effective scale-up, both in low-middle income countries and in high-income countries.

## Background

Heroin use in South Africa has gone mostly unnoticed, mainly due to the lack of robust prevalence data and the ‘rebranding’ of heroin using local names [1]. There has been a slow realisation that drugs locally termed ‘spices’, ‘whoonga’, ‘unga’, ‘sugars’ and ‘nyaope’ all have heroin as the primary active ingredient [2]. Further, people who use drugs are criminalised, heavily policed and are generally not considered as deserving of attention and specialised services [3, 4]. Despite the lack of data on the number of heroin users, increases in trafficking have been documented [1] and the number of people accessing treatment for heroin use disorders over the past two decades has increased [5]. The increases in trafficking and treatment admission, combined with anecdotal evidence, press reports and personal experience, strongly suggest a rapid and significant rise in the use of heroin in South Africa.

Despite the rise in heroin use and an increase in injecting use [1], there is no institutional framework to deal with the public health crisis that predictably follows the increased dependent use of unregulated opioids [6]. Opioid agonist therapy is the gold standard for helping people resolve heroin dependence [7–9]. Methadone and buprenorphine are recommended medications for opioid agonist therapy [10]. Methadone in the right doses and prescribed as a maintenance therapy decreases heroin use, reduces injecting, improves health, reduces HIV and hepatitis C infections [11–14], improves social function and reduces criminal activities [15, 16].

The scale-up of opioid agonist therapy in lower- and middle-income countries has been hampered by political, moral and cultural opposition to this approach [17, 18]. In countries (including in Africa) where opioid against therapy has been made available, the approach has often been regimented and authoritarian and has taken place primarily within medical settings. This has negatively affected retention and therefore ultimately its effectiveness [8, 19, 20].

### Factors influencing retention in opioid agonist therapy

The benefits of opioid agonist therapy become more noticeable over time [21, 22], which is why retention in such programmes is a significant indicator of success. Opioid agonist therapy is strongly advocated by those who adhere to a harm reduction approach to drug use. This approach focuses on reducing drug-related harms [23] rather than on enforcing a prescribed outcome. Treatment goals in a harm reduction setting could range from abstinence to a reduction or more structured or safer form of use. When dealing with opioid use, treatment goals can be aided by opioid agonist therapy. The success of opioid agonist therapy in reducing the harms associated with drug use is significantly linked to retention in such programmes. Important structural, programme, relational and social factors that influence retention are summarised below.

Structural factors, like geographical location, economic conditions, employment, legal status and housing, are more predictive of initiation and retention in opioid agonist therapy [24, 25] than individual factors. Low-threshold programmes facilitate higher retention as they are designed to reduce barriers to entry (including eligibility criteria), are patient-centred, and take a non-punitive approach to drug use and support people in situations of structural precariousness [26–28].

Programmatic factors include dosing, duration of services, programme design and philosophy and standard operating procedures [29]. Changes in retention according to dose are well documented, and methadone maintenance doses should range between 60 mg and 120 mg [30] to optimise effect and retention. Take-home doses increase retention rates because they allow for autonomy, flexibility, and social integration [15, 31, 32]. Retention is further enhanced in the presence of optimal counselling and patient involvement in decision-making, non-punitive approaches to illicit drug use and realistic expectations of employment [33]. Contingency management using incentives has also been shown to improve retention [34, 35].

Individual factors, such as personal characteristics, are often viewed as important predictors of retention. In some studies, younger age [21, 36] and poor motivation predict lower retention. However, there is little critical evaluation of the ‘why’ there is low motivation in the first instance, nor of the ‘sense-making’ that the individual attributes to the attractiveness or appropriateness of the service. Significant literature does seem to indicate that individual characteristics are less important than the counsellor or project characteristics in predicting the resolution of dependent drug use and retention rates in opioid agonist programmes [37–39].

The existence of therapeutic relationships is one of the best predictors of favourable outcomes. Retention is enhanced in the presence of mutually respectful, non-stigmatising, trusting and collaborative relationships [20, 40, 41]. In healthy therapeutic relationships, clinical staff focus on problem-solving as a joint venture with participants when challenges emerge and interactions are bi-directional. When a harm reduction approach underpins the framework of an opioid agonist therapy service, there is increased potential for therapeutic relations to take hold. This increased potential is because ‘harm reduction as a philosophy shifts the moral context in health care away from the primary goal of *fixing individuals* towards one of *reducing harm*’ [42]. In achieving this, harm reduction opens opportunities for promoting the health of people who often are stigmatised through social responses to problematic substance use by not imposing predetermined (and often unrealistic) expectations and outcomes on clients.

Social cohesion (or solidarity) refers to an emotional and behavioural commitment to a group. The humiliation and discrimination experienced by people who use drugs often result in ties based on a shared identity of ‘abnormality’. A closed circuit of trusted ‘comrades’ [43] surviving in stigmatising social contexts emerges amongst those who identify as people who use drugs [44–46]. To draw on the strengths of this social solidarity, many group psychosocial interventions have the potential to enable members to encourage and support one another to optimise treatment outcomes [47, 48]. Social bonds amongst members of the community of people who use drugs have been used to create viable programmes, mainly to prevent HIV infection through needle and syringe programmes [49]. There has been some examination of cohesion in ‘recovery groups’ linking cohesion to satisfaction [50], and self-efficiency [51]. Yet, we did not find any published literature that spoke to social cohesion within opioid agonist therapy programmes. We found no reference to social cohesion as an explanatory factor for retention rates. We believe this to be a serious lacuna given its importance in other treatment programmes and also given the social bonds that are often very apparent (even if conditional) within the drug use community.

### South Africa’s first low-threshold opioid agonist therapy project

Opioid agonist therapy has not been scaled-up in South Africa. Neither methadone nor buprenorphine is included in the essential medicine list for opioid agonist therapy. The National Department of Health guidelines to manage opioid use disorder are currently being drafted, but there is no guarantee that this will be rolled out in the public sector in the near future, despite the drafting of an implementation plan for opioid agonist therapy in the public health sector. Currently, across South Africa ‘treatment’ programmes are mostly abstinence-based. Opioid agonist therapy is predominantly (and nominally) accessed in the private sector and is unaffordable for most people who need it [6, 52].

Within this context, we established South Africa’s first opioid agonist therapy demonstration project in the port city of Durban. This demonstration was primarily designed as an advocacy tool with the intention of showcasing the quality of life improvement amongst low-income heroin users on opioid agonist therapy. The project was located at a discrete drop-in-centre in Umbilo, an accessible working-class inner-city suburb. Methadone was provided to eligible, consenting participants under medical supervision by a team consisting of a part-time general practitioner, a nurse, a social worker and a counsellor. One of the key reasons for using methadone was that it was provided free of charge by the pharmaceutical company for the purpose of the demonstration project, over an 18-month period.

Inclusion criteria included aged 18 years or older, ≥ 12 months history of heroin use with a World Health Organization Alcohol Smoking and Substance Involvement Screening Test (ASSIST) score of ≥ 27 [53], confirmed recent use of opioids through urinalysis, no pending court case, ability to attend daily clinic visits, a person who could provide support outside the project, had stable accommodation for the past 3 months, agreed to be contacted for follow-up and provided informed consent. To minimise risks of overdose and clinical adverse events, people with an acute alcohol or benzodiazepine use disorder, a psychotic disorder or a history of severe head injury, cardiac, respiratory or liver condition were excluded. ECGs were included to identify people with potential cardiac problems at baseline and to gather data on changes over time, following the South African guidelines for the management of opioid dependence developed by the South African Addiction Medicine Society, which recommends baseline, 1 month and annual ECG assessment.

Participants came to know about the demonstration project through word of mouth on the street. Overall, 54 people were recruited over 6 months. The sample size was based on resource availability with a focus on people who smoke heroin (80% of the cohort), reflecting the modes of heroin use in the city and the country [5]. All participants signed a contract which outlined how the project would run and the prerequisites for participation.

The project adopted a harm reduction approach [54] and was designed to be low threshold. Apart from initial screening to confirm opioid use, there was no biological screening for drugs unless clinically relevant. Staff tolerated concurrent poly-drug use unless there was a significant clinical risk. There was no expectation of participation in psychosocial components. The medical team initiated take-home dosing as soon as practical. Participants received 18 months of prescribed methadone as per guidance provided by the South African Addictions Medicines Society [55], particularly in terms of initiation dosage. Although flexible dosing was offered, the average maintenance dose was 55 mg, below the recommended 60–120 mg. While the stabilisation dosage might appear low as compared with other opioid agonist treatment programmes compared with other opioid agonist treatment programmes, the dosage was adjusted in a supervised manner by the medical doctor based on levels of comfortability reported by the participants.

Most participants were male (96%) and most where Black African (63%), with a median age of 28. An overview of participant characteristics and heroin use practices at enrolment are provided in Table 1. The poor take up of females in the project was noted as significant but has not been fully researched. The increased stigmatisation of problematic drug use amongst women in South Africa, and consequently, their reduced health-seeking behaviour seems to be the most likely explanation.

**Table 1:** Baseline characteristics and heroin use practices of clients enrolled in Durban opioid agonist therapy project (*n* = 54)

	Number	Percent
Sex		
Male	51	96
Female	3	4
Age		
Median (interquartile range)	28	25–32
Race		
Mixed ancestry	3	6
Black African	34	63
Indian ancestry	3	6
White	14	26
> 10 years drug use history	30	56
Used heroin > 4 times per day	31	57
Current pattern of heroin use for > 6 months	47	87
Injected heroin in last year	10	19

Participants were initially seen daily for supervised dosing, with slow up-titration until a stable, optimal individualised maintenance dose was reached. Once stable, participants were seen monthly by the doctor and by a nurse at each dosing visit. After 3 months, participants were considered for take-home doses (moving from weekends to longer periods of time). Take-home doses were generally provided to an identified support person. Support people were educated around methadone and the project and were crucial in ensuring that doses were collected from the drop-in centre and taken as prescribed. Participants who missed three consecutive doses had doses reduced as per clinical guidelines. Participants who missed more than 30 consecutive doses were considered lost to follow-up. Participants who were lost to follow-up were not excluded from the project. Instead, they were encouraged to re-join the project, following a medical assessment and discussion with the clinical and psychosocial team. Retention at 6 months was 81%, with a significant reduction in heroin use and a significant improvement in mental health [56]. At 12 months, retention was 74%, with notable improvements in participants’ quality of life [56].

This paper aims to explore the factors underlying the high retention rate and develop recommendations for drug policy development and programme architecture.

## Methods

To gain a nuanced understanding, we used several different qualitative research methods, including semi-structured interviews (*n* = 30), focus group discussions (*n* = 2), oral histories (*n* = 10) and ethnographic observations. Data was triangulated to provide rich descriptions and enable nuanced interpretation of the information obtained from the various methods.

A qualitative research team was established, based at the Urban Futures Centre at the Durban University of Technology. A qualitative researcher, Sibonelo Gumede (SG), conducted the in-depth interviews, focus group discussions and completed ethnographic observations. SG was the same age as the majority of the participants and is fluent in local languages (English and Zulu). The qualitative research was overseen and analysed by Monique Marks (MM), an established qualitative research professor with a doctorate in sociology and a trained social worker. Anna Versfeld (AV), a senior social scientist with a doctorate in medical anthropology and well-versed in conducting participatory research with people who use drugs, co-facilitated the focus group discussions. Andrew Scheibe (a medical doctor with a decade long experience of harm reduction health service provision in South Africa) and Shaun Shelly (an experienced substance use treatment programme and policy advisor) worked with MM to design and monitor the project and to interpret the results of the qualitative data. Strong bonds of trust existed between the researchers and the participants, augmenting transparency in conversations and interactions and thereby enhancing the validity of the research.

The semi-structured interviews explored participants’ pathways into drug use and the opioid agonist therapy project, their meaning attributed to methadone, the factors contributing to project success and changes they experienced in their daily lives. These interviews were done at different times—ten at baseline, ten around the 6-month point and ten around the 12-month point. Interviewees were self-selected, based on willingness to participate. Interviewees were diverse in terms of age and their consistency in the programme. People who injected heroin were included in each of the 6-month interview cohorts. Interviews were not intended to be longitudinal, and as a result, only some of the interviewees were interviewed more than once. SG conducted the interviews, which he recorded and transcribed. SG and MM developed a coding system using Nvivo for the interview data.

Focus group discussions took place around 8 months after the project started. These groups allowed participant to share ideas and perspectives about being part of the demonstration project. The focus groups also provided a forum for them to reflect on their experiences and to vocalise these reflections. Focus group questions were centred on reasons for participation in the study, experiences of being on methadone and factors that contributed to retention. Focus group participants were active in the mindfulness groups run by peers and the psychosocial team. One focus group consisted of ten participants and the other of seven. SG and AV facilitated the groups in English and Zulu. Written notes were taken while the focus groups were being conducted. These were also coded using Nvivo.

MM conducted oral histories at around the 12-month interval. The reason for this time interval was that at this point, participants had been on the programme for a considerable period of time. This time interval also allowed for the identification of participants who had opted out of the programme to be identified and interviewed. Their stories were viewed as equally important in making sense of their life journeys and their choices in regard to their drug use. Oral history interviews were unstructured and provided participants the opportunity to tell their life histories in a manner they were comfortable with. Oral histories allowed for an in-depth exploration of participants’ life circumstances (historical and present), pathways into drug use and into the project. MM approached oral history participants with the intention of selecting participants with various drug using practices (including three people who injected heroin), a range of self-determined goals (ranging from abstinence, to maintenance on methadone, to reduction in heroin use) and differing lengths of time on the project (including two people who prematurely exited). These oral history interviews lasted between 1 and 3 h. The oral histories were recorded but were not transcribed at the time of writing this paper. MM provided data from the oral histories through re-listening to the recordings. The general focus of the oral histories aligned closely with the focus groups and with the interviews. The more informal and self-determined approach of this method allowed for a telling in a manner that was therapeutic and often bi-directional.

Throughout the project, observational research was conducted. Field notes were written by SG and were coded deductively using Nvivo. These codes were informed by what had emerged in the interviews and focus groups. SG participated in all aspects of the participants’ life in the project—playing soccer, joining mindfulness groups, spending time at the drop-in-centre and visiting some participants in their homes. He, together with other project team members, attended court cases when participants were arrested in the early stages of the project’s lifespan. Observations focused on interactions between participants and between participants and project staff, and also on observable quality of life changes. More contained observational research was conducted by two external researchers. Michael Wilson, a public health specialist from the USA based in Durban, spent substantive amount of time at the project site. He was not a passive observer, but rather worked with participants to develop a photo essay, which was welcomed by participants. A second outside observer, Prof. Dustin Patil (Director of Addiction Psychiatry at Tufts Medical Centre, USA) spent 2 days at the drop-in-centre. The participants were informed of his wish to visit and observe the project. Participants provided their consent for him to visit as they were keen to engage with him about similar projects in other parts of the world. After his visit, Prof. Patil sent notes of his observations to MM.

In this paper, we draw on the various qualitative data elements to make sense of the factors contributing to retention. Significant time was spent analysing the various qualitative data sources and triangulating them. We reviewed the primary data from the qualitative research in light of the existing literature on opioid prescribing, programme effectiveness and retention rates. In so doing, we were able to develop an exploratory explanation regarding the relatively high retention rate in the project. The research team had several analytic conversations about the findings. We engaged with participants about what they felt had contributed to their retention in the project and shared our analysis with them, thus allowing for member checking. This member checking was done both individually and in group sessions.

The Durban University of Technology and the KwaZulu-Natal Department of Health research ethics committees provided ethical approval. Participants provided informed consent, and we have used pseudonyms when quoted. Participants did not get paid for participation.

## Findings and discussion

The retention rate of the project at the 1-year interval was 74%, higher than the global average and significantly higher than our initial expectations as the project team of around 50%. In this section, we explore the two most important factors we identified from the qualitative research conducted as contributing to the outcome: the philosophy and architecture of the project, and social cohesion. Programmatic factors were more significant than individual or structural factors in determining this retention rate.

### The philosophic and programmatic architecture of the project

The principles of harm reduction and a low-threshold approach informed the design of the project; we aimed to attract participants by meeting their needs rather than through coercion. The philosophy of the project was not to ‘treat or cure’ the individual; the goal was to empower people, to make well-informed conscious decisions about their use or non-use of drugs. Motivational interviewing, collaboration and unconditional positive regard informed the way staff interacted with participants. In interviews (including oral histories) and in focus groups, participants spoke about the importance of being able to engage honestly, without fear of recrimination, to the project staff. As a 27-year-old Sanele put it in an oral history interview:

I think for most of us, this is the first time we have been treated as human beings. We have always been discarded by society. In this OST [opioid agonist therapy] project, we do not feel looked down upon. We have been able to start to respect ourselves and feel positive about our futures. We also feel free to be honest about what is happening in our lives, even if we are using. This is different from other rehabs I have been to where you are expected to be clean.

Kevin (31 years old) expressed similar sentiments:

My whole life I have felt dislocated and sort of uncared for…I started using drugs when I was about 12. My father had been arrested, and my mother was working two jobs. My whole family has shunned me except my mother, although I have made her life difficult. I had no hope of coming right before the OST [opioid agonist therapy] project. Here I felt I was treated like a person with dignity. After being on the project for a few months, I looked at myself in a mirror – right in the face – for the first time in 10 years. I felt cared for and unjudged, and so I was able to stop being so hard on myself as well and just find where I need to be rather than be told what I should be doing.

What participants, like Sanele and Kevin, expressed in various forms of interviews (in-depth and oral histories) was that being able to set their own goals and feeling free of judgement created an internal sensibility of wanting to be part of the project and to return daily. Even when participants missed doses or were considered lost to follow, they knew that they were welcome to return. In interviews, focus groups and oral histories, a consistent theme that emerged was the importance of having a ‘safe space’ to talk about their drug use histories, and their periods of ‘relapse’ or absence from the programme. For participants, the lack of condemnation in regard to both of these experiences allowed for an honest interaction with project staff and with one another. This approach is contrary to what most had experienced in other treatment interventions. This openness and lack of judgement, in their view, was the key to their desire to participate in the project and to spend significant amounts of time with their peers and with their project staff. Simply showing up at the drop-in-centre as a safe space facilitated retention as this was where dosing was done.

The physical space that housed the project reinforced feelings of belonging and dignity. A former garage was provided to them, which they transformed into a lounge area. They painted this lounge space in bright colours, and the words ‘home away from home’ was spray-painted on the walls. Many participants spent almost entire days at the drop-in-centre engaging one another, having group sessions, writing and reading, and huddling together to watch movies on a small computer screen. They had access to computers which they used to develop their CVs, find work, check emails, and apply for study courses. Participants developed their own set of ‘rules’ for engagement while at the drop-in-centre. These were informed almost instinctively by two thought paradigms—harm reduction and restorative justice. Combined, these provided a strong sensibility of unconditional acceptance of one another’s humanity and flaws, while at the same time anchoring this within an imperative to act responsibly towards others. Drawing from the work of Bourgois, we could say that participants were ‘in search of respect’ [43]. They found this, for many for the first time, in the project. This sentiment emerged consistently in interviews, oral histories and in the focus groups. Observational recordings testify to the daily interactions at the drop-in-centre and to the care and freedom with which participants spoke about all aspects of their lives to their peers and to the project staff.

The philosophic and programmatic architecture contributed to the high retention rate, as is evidenced in generalised sentiment amongst participants. However, there was another critical factor that emerged as perhaps the crucial contributor to this high retention rate—social cohesion amongst the participants.

### Social cohesion

The project brought to the fore the importance of social cohesion (or solidarity) in keeping participants in treatment. Extraordinary bonds of care, acceptance and mutual support formed quickly amongst this group. This level of cohesion would, for many, be unexpected as the group itself was not homogenous. The participants did not know one another before joining the project, and their substance use goals differed widely. While most people were in their late 20s, there were three men in their forties and two were still attending high school. Religious diversity ranged from Christianity to Islam, to Hinduism and African traditionalism. The group was representatively racially diverse, and home languages included Zulu, English and Afrikaans. The group had widely differing goals in respect of their drug use. These goals ranged from reducing heroin use, to methadone maintenance and to total abstinence. Despite these differences between participants, through their often raw interactions with one another in peer led groups, what emerged was how intertwined their stories were. This was particularly in regard to their negative interaction with the criminal justice system and their shared view that previous ‘rehabilitation’ experiences had failed to assist them in normalising their lives. This project provided a platform for participants to change the narratives of their own lives and of treatment.

Participants, when asked in interviews and in the focus groups about their reasons for staying in the programme answered that a crucial factor was the feeling of unity and camaraderie within the cohort that spurred them on. This spirit de corps was observed by public health specialist, Michael Wilson, at a talk he gave at a Drug Policy Week conference held in Cape Town in October 2018:

When I started to spend some more time in this centre, and getting to know some of men and women, observing their interactions with one another and with the staff and actually getting behind the camera, I realised this community is really unique in ways that I hadn't seen before…There is a sense of deep respect and transparency and camaraderie among participants. There is an eagerness to see each other be the best. There's a spirit of sharing, whether its water from the fountain, cigarette or food money. There is a desire to volunteer and to make their community into a better place, and there's a sincere interest in each other and in each other’s goals. They’re using the community they built with one another, the knowledge that they’ve acquired, and the confidence that they’ve built, as a real launching pad to achieve truly honourable and significant life goals.

This was very different from what he had observed in opioid agonist therapy programmes he had been involved with in the USA and in Haiti. His views were shared by project participants whom he engaged with over a period of around 6 months through a photographic project. No doubt the provision of free methadone provided significant incentive to visit the centre each day during phases of observed daily dosing, but this does not account for the fact that participants would spend entire days there, even after take home doses were initiated.

By sharing stories and through joint activities, including group sessions, running a peace committee (described later) and engaging in sports and volunteer work, bonds were formed and reinforced. They had all suffered the consequences of social exclusion and the project provided a refuge from this. By example, after Muzi expressed his negative experience of growing up in a foster home, more participants started speaking out about their traumatic experiences. They reached out to one another both physically and emotionally. The physical expression was through simple touch and through the sharing of scarce resources such as money or cigarettes. A 20-year-old school going Vuyani provides a beautiful explanation of what the group cohesion meant to him:

I joined the project not sure if I wanted to be there. But then when I joined the project, I met the other guys. Now I would say they are brothers to me. When things have gone really bad, they have supported me. When I didn’t take my doses for a few days, they came to find me at my home and see what was happening. Knowing that they cared so much made me want to make them proud of me…In the past, when I have tried to stop drugs, I have felt all alone. Now I know that I am not. Of course, I come to Umbilo to get my dose, but I also come to just be there with the guys, knowing that we are always there to encourage each other.

What Vuyani describes is mutual inter-dependence and a sense of belonging. This belonging spurred him on even when he was uncertain of his commitment to the project. There were many instances when the group ‘showed up’ when participants were about to give up. They visited one another’s homes or looked for each other in places that they knew they were likely to visit (or inhabit).

The sense of connectedness that was so significant to participants in their reflections on the project was also evident to Prof. Dustin Patil. In an email to Monique Marks after his visit, he relayed his amazement at the difference between the Durban projects as compared with any other like projects he had interfaced with.

As a foreigner accustomed to the medicalized, “dose-and-go” Opioid Treatment Projects of the United States, I was struck by the sense of community among participants at the Durban project. To me, the Durban project felt more like a clubhouse than a methadone clinic. Individuals sat in a communal room with arms slung over one another’s shoulders; they spoke like old friends and referred to one another as “brothers.” They shared stories about the life-changing benefits provided to them by the project. They empathized with each other’s hardships and celebrated each other’s successes.

The Durban project provided much more than medication to its participants. Individuals found a culture of connection and belonging that I have yet to encounter at another opioid agonist therapy project.

For project participants and those observing the project, the social cohesion within the group was significant and unique.

Given the centrality of this cohesion to the participants’ understanding of the ‘success’ of the project, we felt it worthwhile to try to identify what collective activities fostered this. Identifying these is not only important in explaining what emerged in this project, but also in providing possible pointers for crafting a similar environment in other opioid agonist treatment programmes both in South Africa and globally.

After analysing the qualitative data, we identified group sessions, the peace committee and group sports as critical contributors.

### Group sessions

Two types of group sessions took place during the project—peer led and professional led. In both groups, participants were encouraged to set their own goals and to talk openly about any challenges they were experiencing, particularly regarding attaining their self-determined goals. Participants noted that in these groups, they could speak without fear of judgement or punishment, but with the knowledge that every experience they have brings with it essential learnings. Participants referred to the groups as crucial spaces for them to explore individual dilemmas and hurts and for assisting one another with goal setting and goal attainment.

As researchers, we observed that participants drew on cultural practices from ‘home’. Most participants grew up in African townships where a belief system and tradition of universal bonds of sharing exists and connecting to others as fellow humans exist. This is best captured in the Nguni Bantu word ‘ubuntu’ which literally means ‘I am because we are’. Embedded in this organic philosophy and practice is a belief that universal bonds connect all humans, and this compels behaviour that is compassionate, reciprocal and mutually caring. This Ubuntu was evident in the group, and participants developed sets of practices of sharing and bonding that could be viewed as organic. Encouragement and support, which emerged in very spontaneous ways, took the form of verbalising praise, saying poems, clapping and even gifting. Tears flowed when stories were shared, and participants found ways of creating holding spaces when this occurred. Groups became healing circles as participants spoke about their bitterness and shared their triumphs. While all respondents came from resource-poor backgrounds, they shared what they had with one another.

A 25-year-old man whose life shifted dramatically from living on the streets to living at home and applying for tertiary studies, when interviewed, spoke about the importance of sharing life experiences in the group sessions:

…hearing other people’s problems helped me assess my own problems and realise they are minor compared to other people’s issues. They helped me change my mindset from focusing on my problems, which drove me to smoking [heroin] but rather created an eagerness in me to change and learn from my mistakes.

Instead of competing in the group, what emerged was a collective claim on what might otherwise be understood as individual achievement. Siyanda, a 24-year-old who had lived on the streets for 5 years before joining the project, commented that:

The group means everything to me. I call them my brothers and sisters. We learn from each other, but I also feel encouraged when I see that other people are achieving their goals. It is not like on the streets where if someone is doing well, you try to take what you can from them. Here we are all in this together. The group sessions allow us to help each other and to work through the problems we are having.

We should not romanticise Ubuntu, nor simplify how cultural practices can be leveraged to enhance project outcomes. Participants shared in the interviews and in the oral histories how they entered the project with scepticism, but that the environment created by the project team (physically and psychologically) allowed for emergent practices of Ubuntu to flourish. In this project, the solidarity between the participants extended from the parameters of being fellow drug users to seeing one another as family, resulting in positive spinoffs in terms of harm reduction outcomes. A sense of collective pride in personal hygiene and wellbeing quickly spread amongst group members and the sharing in groups allowed for psychological healing and sometimes an economic boost (nominal as it was) in material circumstances.

While the groups were in progress, participants pooled their resources. They shared cigarettes, cold drinks, food and money. They created collective funds for achieving shared aspirations, such as registering for a learner’s driving licence. These collective funding schemes were modelled on institutional arrangements of co-operative pooling of funds in the townships called ‘stokvels’[69]. These indigenous values were diffused from township practices into this inner-city site, demonstrating how socially disregarded spaces are perhaps where the greatest (and best) learnings take place and can be transferred into other settings. This sharing, in a resource-poor community, reinforced social bonds.

### The peace committee

At the beginning of the project, few participants were familiar with the level of trust they were afforded by staff, and a range of anti-social behaviours were exhibited. Stealing occurred frequently and affected project staff whose mobile phones and laptops were taken. Chairs, tables and basic kitchen items went missing and had to be replaced. Two participants had episodes where they displayed an inability to contain their anger and frustration. These incidences induced fear amongst project staff and fellow participants and required careful psycho-social intervention.

In the first 6 months of the project, the group formed a ‘peace committee’ in an attempt to address antisocial behaviours. Following restorative principles [57], the peace committee brought project participants together to resolve conflicts or to find solutions when harmful or hurtful events had taken place. The peace committee mirrored those that were established in South African townships in the 1990s. These township-based peace committees were initially formed to find restorative ways to deal with community-based issues which would otherwise be dealt with by the state criminal justice system. Township peace committees brought together community members to get conflicting parties to address the structural problems underlying acts that offended the collective [57].

When participants heard about the peace committees from one of the project team leaders, they decided to establish one themselves, although there were a few participants who were sceptical. With the assistance of an expert facilitator from Cape Town, John Cartwright, the Durban group formed a peace committee and adapted the processes and structure to fit the context of the project. However, the underlying principles were the same, i.e. that peace-building and peace-making should be at the core of dealing with community-based conflicts and hurts. Punitive approaches were viewed as inflicting further stigma and disconnect, which in turn would negatively impact on the journey towards life normalisation. When the peace committee was suggested, there were divided views on its usefulness. The majority, however, decided to test out the peace committee.

The peace committee brought together all parties affected by an incident of ‘wrongdoing’ to decide collectively (and consensually) how to deal with its consequences. Such incidences could range from acts of stealing, to minor assault or to hurtful slander. As part of the peace committee process, group members had to find ways to restore peace while getting the wrongdoer to take responsibility for their harmful action. One of the authors who sat in on some of the peace committee meetings noted that they were run with great empathy and understanding. Knee-jerk reactions were not tolerated. Instead, what was encouraged was deliberation and concern with understanding root causes and with reintegrating the ‘wrongdoer’ into the group.

All peace committee matters were dealt with in a restorative manner, emphasising that the person (or persons) who had acted offensively be embraced by the group after having recognised their wrongdoing, conveying an apology to those affected and making reparations where possible. This restorative, non-punitive approach allowed for self-regulation and reintegration. As Siyanda put it:

The peace committee meant a lot to us. It helped us to deal with issues as a group in a way that we did not punish. So by doing this, we did not exclude anybody. We wanted to understand why certain things had happened and try to make things right again. We understood that we all have done wrong things but that we are not bad people. So the peace committee helped in getting people to be responsible but also know that they were not rejected…The peace committee also helped us to cool our tempers when we sometimes felt really angry at someone.

These restorative practices reinforced the broader harm reduction philosophy of the project. We are not aware of other opioid agonist therapy projects that have incorporated similar processes for conflict resolution. It is also worth noting that, in the duration of the project, there were no physical altercations between participants. Disputes or conflicts were handled directly by the peace committee process or by individuals who were nominated by the beneficiary group to be part of the peace committee body. Peace committee representatives were held in high regard, itself testimony to the connective bonds between project participants.

### Group sports

Fairly early on in the project, the group formed a soccer club. This was significant for two reasons. Firstly, it provided a collective recreational outlet while bringing tacit sporting skills to the fore. As a group, they competed with other soccer teams in the surrounding areas, reinforcing group spirit. Those participants who did not play soccer, as well as staff members, would go and watch the team play.

Secondly, playing soccer was another way of building a sense of health and wellness. When participants joined the project, they had very little concern about their appearance and wellbeing. This changed within a short period of being on the project, and participants were almost unrecognisable a few months into the project. They had transformed from looking street rough to looking well presented. Participants took turns to use the shower and laundry facilities available at the drop-in-centre. Self-pride replaced shame, and this was evident in the confidence that the soccer team displayed on the field, and through their supporters. In many of the interviews, it emerged that this new self-care was not just for self-gratification, but was also important because they wanted their families and the rest of the participants to be proud of them. Participants were keen to shed the label that had been forced upon them by their communities of origin and the general public—‘amaphara’ (parasites who sucked resources from communities and families to fuel their drug use). In an oral history interview, a 28-year-old Temba spoke about the importance of the soccer group in his ‘recovery’.

Another thing that helped me was the soccer team. When we started it I wasn’t sure it would work. But then practice become something important to us. It built team spirit, and we had group goals. I can say that it helped us to have a healthy lifestyle as well, which was good for our recovery as a group. I would look at others and see that they are looking healthy, and I wanted the healthy and fit life…Now we don’t look like people from the street anymore.

These collective activities we have outlined above were crucial in forging social cohesion. This connection and to a large extent, the shared identity, did not result simply from moments of contact when participants were at the drop-in-centre. As Valentine [58] pointedly argues, proximity alone does not produce collective identity and connectedness. For this to occur, she argues ‘meaningful contact’ must be present whereby people demonstrate care and interest in one another’s lives. Meaningful contact is not seamless but is interrupted by discomfort and intolerance and then remedied once more through a deepening contact characterised by interdependence and care. Observations and interviews revealed that moments of rudeness and confrontation occurred precisely because of proximity. However, equally evident was that these interruptions were temporary, outweighed by a recognition amongst the participants of their current situations and their shared goals to reduce harm and to normalise their lives under challenging circumstances. And collective activities such as the peace committee reduced potentially harmful experiences between participants and with project staff.

## Limitations

As architects of this project, we had a vested interest in its success. While the retention rates cited have a numerical ‘objectivity’, our embeddedness in the lives of the project participants and our advocacy role might have influenced our analysis of the factors contributing to the project’s accomplishments. Having said this, rigorous qualitative and quantitative data was collected throughout the demonstration project. This paper only refers to the qualitative data set. Given the possible advocacy bias, it was valuable to receive input from external researchers in making sense of the high retention rate.

The majority of participants were smokers rather than intravenous users. This is unusual for methadone projects, particularly in Africa, that have historically been implemented to prevent infectious diseases, most particularly HIV. The logic for this selection was sound given that the vast majority of heroin users in South Africa are smokers. While the sample was small, proportionately fewer people who injected were retained in the project compared to people who smoked heroin; had the proportion of smokers to injectors been inverted, results in relation to retention might have been notably different. However, given that main modality of use in South Africa is smoking rather than injecting heroin, this study’s outcomes and findings are valuable outside the city, in South Africa more broadly, and in other contexts where heroin is mostly smoked.

Another limitation was the exclusion of participants who were homeless and those with serious psychiatric conditions and co-occurring substance use disorders associated with an elevated risk of overdose. In other words, some of the structural factors known to contribute to retention rates were eliminated from the study. Given that this project was in many ways developed as an advocacy tool, the architects of the project tried to design a project with a lower risk for participants. All participants were drawn from low-income backgrounds, and some of the housing arrangements would be considered ‘informal’ in other country settings. It is worth noting that there were a few homeless participants in the project. They entered by providing physical addresses that they were not currently living at, and at a later point, they revealed their status as homeless. Interestingly, but perhaps not significantly, the few homeless participants were retained to the project endpoint and while accessing the opioid agonist were achieving their self-determined goals.

As this study did not include a control group, we have no means of knowing if the results would have been different without the factors that led to the levels of cohesion described. We do not know, for example, what the retention rates would have been had the majority of participants been homeless or if there were no support persons. What is clear is that the group of participants and the observers consistently identified the level of cohesion as a significant contributor to the positive outcomes noted. This emerged organically and was not consciously designed into the programme, but there are programmatic implications of this study for opioid agonist therapy which we discuss in the next session.

## Conclusion and practice implications

We believe that the project in Durban is fundamentally different when compared to opioid agonist therapy programmes in similar settings, i.e. in poorly resourced contexts with low-income participants. Typically, programmes emphasise the need to control the dependent service user through daily contact, supervised dosing on an ongoing basis, drug testing, emphasising abstinence and enforcing a shared set of common, fixed goals that are non-negotiable. We believe that we achieved high retention by using an approach that attracted participants by meeting their needs and minimising barriers, as opposed to creating a sense of obligation arising from group pressure or by explicit or implied coercion. Being treated with dignity, as people who use drugs, often for the first time, was a consistent theme across all data sources. The vast majority spoke of the ‘failure’ of the punitive and directive approaches common to most rehabilitation programmes. How exactly programmes design bi-directional care and respect is fundamental to the provision of effective healthcare, particularly for highly vulnerable groups of people, and needs to be given careful consideration when hiring staff and engaging peers in service delivery.

Having a support person to assist with take-home doses can be critical, not only to prevent overdose but also to provide a link between the beneficiary and the service provider team. Given their oversight and care role, having an identifiable support person can prevent the common, but often misguided, concerns around diversion. In less developed countries (and even in low-income or remote communities in high-income countries) where access to health care facilities might be limited due to lack of transportation, establishing an arrangement for support and monitoring in the home setting is an important consideration.

Our data suggests that the bonds of care amongst the participants and between participants and the health/psycho-social team stood out as the most important contributor to retention. The connection or social cohesion evident amongst participants was not conditional in the way that it sometimes is amongst the drug use community, where solidarity can be about basic survival. Recognising the importance of mutual individual responsibility, together with interdependence, generated a sensibility of consistently striving to create and maintain supportive and non-judgemental social bonds of care and meaning.

The everyday acts of kindness, empathy and sharing were part of the narrative provided for wanting to remain in the project despite the external stigma of being on methadone. Meaningful contact, while in this case organic in many ways, was fostered through deliberately engaging in positive collective activities and in consciously working against past experiences of stigmatisation, exclusion and disregard. What this means is that social solidarity can (and arguably should) be cultivated through developing programmes that build collective identity. This study provides three examples of how this can be done drawing on the Durban demonstration project. There are undoubtedly many others that could be activated that fit culturally and contextually in other settings.

Our research shows that programmes that encourage social cohesion can increase the effectiveness of opioid agonist therapy. The data shows that social cohesion can be achieved by paradoxically embracing and accepting the differences between the members rather than enforcing ‘group think’. Restorative processes, such as those used by the peace committee, for dealing with conflict or disruption align with the principles of harm reduction, improve outcomes and should be incorporated into programmes.

Group identity, pride and support have the potential to play a decisive role in retention. The question that we should ask is why there is an absence of translating this feature of group solidarity into making sense of treatment settings, and more specifically of retention rates. In addition, we should be asking how social cohesion can be developed in projects such as this one. Given the findings of this study, we recommend that all health professionals receive training that emphasises the need for and capacitates them to develop bi-directional and non-authoritarian relationships with service users. On-site activities that foster social cohesion are equally important and should be considered as part of the programme architecture. These include peer-led groups that combine mindfulness with mutual support, group activities that are determined by service users and restorative mechanisms for dealing with harmful and hurtful behaviour enacted by both staff and service users in any such programme. These should not be viewed as fringe activities in an opioid agonist therapy programme, but rather as foundational to realising treatment goals.

There is, however, no cookie-cutter method for achieving social cohesion. Instead, what needs to be recognised is the positive potential of cultural capital and emergent practices of people who use drugs. There is often the presumption that people who are on methadone have limited internal resources and are lacking in willpower or agency to initiate and sustain change. We have found the opposite; if the services offered acknowledge autonomy and encourage agency, promote a collaborative approach, respect people’s choices and provide a welcoming, non-judgemental space that is informed by the group and their contextual needs, people will engage with services and prioritise their participation. As this paper demonstrates, cultural capital may form the basis upon which solidarity and restoration are built. Opioid agonist therapy service providers would be well advised to identify and mobilise the cultural capital and the almost universal desire to belong, to seek out supportive relationships and to find ways of utilising them in further embedding harm reduction and restoration. For example, mapping exercises that focus on coping strategies should take place early on in these programmes and deliberations should be held with participants as to how they can use these to benefit themselves and their peers.

To conclude, this project demonstrates that social cohesion is a key factor in attaining high retention rates. The limitations of this study, particularly with regard to the study cohort (primarily smokers) and selection criteria (such as stable housing), may suggest that this study is not generalizable to opioid agonist therapy programmes that do not have the same entry requirements. We contend that these outcomes do have relevance to like projects in both the global south and the global north that target low-income participants. What is required is more support and buy-in for pilot studies such as this one, particularly in the global south. As part of these pilots, qualitative and quantitative research should be built into the pilots to further explore the role of social cohesion as a catalyst for retaining participants in opioid agonist therapy programmes.

## Data Availability

The interview datasets generated during this project are not publicly available due to confidentiality reasons and the diverse nature of the material spread across both analogue and digital storage. Source data can be discussed with the corresponding author and will be supplied upon reasonable request.
